# Spatial Attention Modulates Spike Count Correlations and Granger Causality in the Primary Visual Cortex

**DOI:** 10.3389/fncel.2022.838049

**Published:** 2022-06-16

**Authors:** Qiyi Hu, Zhiyan Zheng, Xiaohong Sui, Liming Li, Xinyu Chai, Yao Chen

**Affiliations:** School of Biomedical Engineering, Shanghai Jiao Tong University, Shanghai, China

**Keywords:** spatial attention, primary visual cortex, non-overlapping receptive fields, spike count correlation, Granger causality

## Abstract

The influence of spatial attention on neural interactions has been revealed even in early visual information processing stages. It resolves the process of competing for sensory information about objects perceived as targets and distractors. However, the attentional modulation of the interaction between pairs of neurons with non-overlapping receptive fields (RFs) is not well known. Here, we investigated the activity of anatomically distant neurons in two behaving monkeys’ primary visual cortex (V1), when they performed a spatial attention task detecting color change. We compared attentional modulation from the perspective of spike count correlations and Granger causality among simple and complex cells. An attention-related increase in spike count correlations and a decrease in Granger causality were found. The results showed that spatial attention significantly influenced only the interactions between rather than within simple and complex cells. Furthermore, we found that the attentional modulation of neuronal interactions changed with neuronal pairs’ preferred directions differences. Thus, we found that spatial attention increased the functional communications and competing connectivities when attending to the neurons’ RFs, which impacts the interactions only between simple and complex cells. Our findings enrich the model of simple and complex cells and further understand the way that attention influences the neurons’ activities.

## Introduction

The brain’s capacity is limited, and the attention can selectively prioritize the goal-related information when multiple stimuli appear simultaneously. In neurophysiological studies of spatial attention, the basic observation is a relative improvement in the firing rates of neurons for attended vs. unattended stimuli in essentially every visual brain area (for reviews, see Buschman and Kastner, 2015; Maunsell, 2015; Moore and Zirnsak, 2017).

In addition to the firing rates, researchers found that the variability in the neural responses to identical stimuli is correlated across neuron populations, which is referred to as “spike count correlations (*r*_*sc*_)” and applied to infer functional interactions (Zohary et al., 1994; Bair et al., 2001; Cohen and Kohn, 2011). Previously reported *r*_*sc*_ are measured from neurons with overlapping receptive fields (RFs), in which small and positive correlations and attention-related decreases have been reported (Cohen and Maunsell, 2009; Cohen and Newsome, 2009; Mitchell et al., 2009; Gregoriou et al., 2014; Ruff and Cohen, 2016). Reductions in *r_sc_* are expected to improve information encoding by decreasing redundancy in signals from neuron populations (Herrero et al., 2013; Moore and Zirnsak, 2017; Denfield et al., 2018). However, the findings in these studies are more likely limited to neurons located nearby or within the same microcolumns. Both anatomical (Gilbert and Wiesel, 1983) and fluorescent imaging studies (Stettler et al., 2002; Liang et al., 2017) have found that neurons with non-overlapping RFs may interact through long-range horizontal connections, which may play a key role in *r*_*sc*_ (Hassen and Hamed, 2020). Unfortunately, very few studies have investigated the *r*_*sc*_ between neurons that were far apart. They observed negative *r*_*sc*_ in FEF (Cohen et al., 2010) and dlPFC (Leavitt et al., 2013), which share differences with the results from pairs with overlapping RFs. So we tried to answer the following question: How does spatial attention influence functional interactions between neurons with non-overlapping RFs in V1?

Previously reported methods that attempt to identify connections between neurons, like cross-correlogram (e.g., Brody, 1999) and joint peri-stimulus time histogram (e.g., Gerstein and Perkel, 1969), provide little insight into the directional nature of the connections and are less reliable to detect inhibitory connections (Stevenson et al., 2009; Kim et al., 2011). Granger causality is an effective method to investigate the causal relationships in sensorimotor, visual areas, and prefrontal cortical networks (Brovelli et al., 2004; Gregoriou et al., 2009; Bosman et al., 2012). *r*_*sc*_ ignore the temporal structure of the recorded spike train, while the Granger causality addresses this limitation and treats the spike train as time series containing intrinsic property (Alonso et al., 1996; Bastos and Schoffelen, 2016). Nevertheless, few studies explored the attentional effect on neurons within V1 by Granger causality. Thus, we applied Granger causality measurement [proposed by Kim et al. (2011)] in our study to further understand how neurons with non-overlapping RFs cooperate in V1 in different spatial attention conditions.

Neurons in V1 can be classified as simple and complex cells according to their response linearity (Hubel and Wiesel, 1962). Previous studies suggest that there is a hierarchical architecture between simple and complex cells (Hubel and Wiesel, 1962; Martinez et al., 2005; Antolik and Bednar, 2011), that is, complex cells would integrate inputs from simple cells (Yu and Ferster, 2013). However, it remains unclear how attention influences the interactions within and between these two groups of V1 neurons when the neuronal pairs are overlapped with different stimuli.

We simultaneously recorded neuronal pairs from V1 with non-overlapping RFs while monkeys performed a spatial attention task to detect the color change. When the attentional focus shifted from the location far away from the neurons’ RFs to the location that covered one of the neurons’ RFs, we found that *r*_*sc*_ between neuronal pairs tend to increase from negative to positive, while Granger causality among them was decreased to negative. This attention-dependent change was consistent only among simple and complex neuronal pairs. We also explored the effects of each neuron’s preferred direction, the factor reported previously to contribute to neuronal interactions (Smith and Sommer, 2013; Liang et al., 2017). Our results indicate that the competition between neural representations of target and distractor relies on the attentional modulation of functional interactions between simple and complex neurons.

## Materials and Methods

### Subjects

All behavioral and electrophysiological data were obtained from two adult male monkeys (*Macaca mulatta*; monkey P: 7.5 kg; monkey S: 9 kg). Animals were housed individually with around four other monkeys on a 12 h of light/dark cycle. All surgeries and experimental procedures conformed to the NIH guidelines and the Institutional Animal Care and Use Committee of Shanghai Jiao Tong University.

### Surgery

Surgical procedures on monkeys were conducted under general anesthesia using the aseptic condition. After premedication with atropine (0.05 mg/kg, intramuscular) to reduce salivation and an antibiotic (benzylpenicillin, 5 mg/kg, intramuscular) to reduce intraoperative infection, monkeys were sedated with ketamine (15 mg/kg). Anesthesia was maintained with isoflurane (2–3%) during the surgery. To reduce postoperative infection, we administered cephalosporin for the next 5 days after the surgery.

To minimize head movement, we first implanted a titanium head post in each monkey before training. Both animals were implanted with scleral eye coils for measuring eye movements by eye-tracking equipment (1,000 samples/s, ScleraTrak 4000, Crist Instrument Co., Hagerstown, MD, United States). After monkeys learned the task (around 6 months), they were implanted with a titanium recording chamber containing the microdrive system (Gray Matter Research, Bozeman, MT, United States) above the dura of V1 in one hemisphere. We determined area V1 by stereotactic coordinates preoperatively (monkey P, right: 15 mm, posterior interaural: 20 mm; monkey S, left: 15 mm, posterior interaural: 20 mm). During the surgery, we used bone screws to fix the chamber to the skull and acrylic cement to seal the opening between them.

### Visual Stimuli and Experiment Task

We presented visual stimuli on a liquid crystal display (1,920 × 1,080 pixels, 120-Hz refresh rate; AOC, Inc., Wuhan, China), which was placed 57 cm away from the monkeys. All visual stimuli were presented using custom software (written in MATLAB using Psychtoolbox-3). After performing the receptive field (RF) mapping (see the “Data analysis” section), monkeys were required to perform a fixation task to determine the preferred direction of the recorded neuron. After a brief buzz at the beginning of each trial, a centrally located fixation point and a pair of sinusoidal gratings with directions of movement uniformly distributed over 8 orientations from 0° to 315° (temporal frequency: 2 cycles/s; spatial frequency: 0.5 cycles/degree; contrast: 90%) were presented. The gratings covered the RFs of recorded neuronal pairs. Monkeys were rewarded for maintaining fixation on the point for 2,500 ms. We chose the direction that evoked the strongest spiking activity as the preferred direction.

**FIGURE 1:**
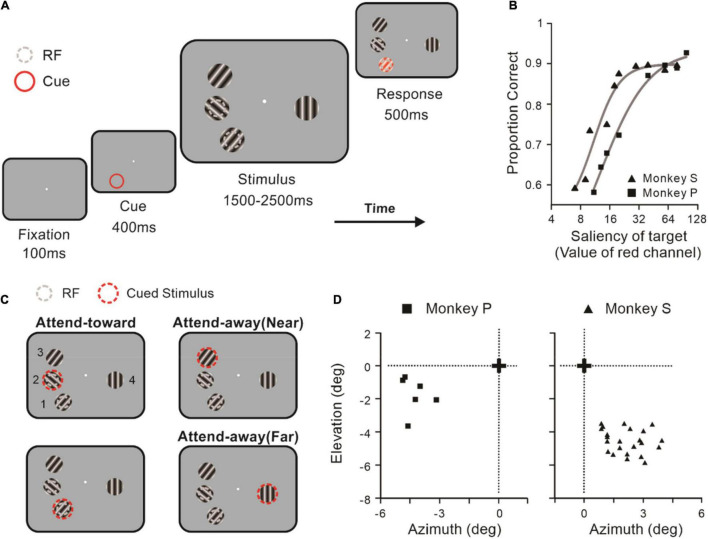
Behavioral task and results. **(A)** The paradigm of the color-change detection task. After monkeys fixated on the white spot for 100 ms, a red ring appeared for 400 ms as a cue for attentional focus. Then four sinusoidal drifting gratings with identical eccentricity were presented at different locations for a uniformly randomized period (1,500–2,500 ms). The monkey was rewarded for the successful saccade to the grating at the cued location within 500 ms after the color of the grating trough changed to red. **(B)** Performance curves of two monkeys across sessions. Individual points represent the averaged detection accuracy at a given value of redness (monkey P: square; monkey S: triangle). Solid lines represent fits of logistical function to the data. **(C)** Illustration of attentional conditions. Two non-overlapped stimuli cover the RFs of the recorded neuronal pairs independently. The dashed red rings indicate the cued stimuli. The condition is defined as an attend-toward (AT) condition when the cued stimuli overlap one of the recoded neurons’ RFs. The cued stimulus appears on the ipsilateral side of neurons’ RFs in the attend-away near (AAN) condition, while it presents on the contralateral side in the attend-away far (AAF) condition. **(D)** The location of RF centers (monkey P: square; monkey S: triangle) of recorded neurons and the fixation point (black cross).

After that, the monkeys performed a color-change detection task (shown in Figure 1A). We used custom software (written in MATLAB using the NIMH MonkeyLogic 2 Toolbox) to present visual stimuli and monitor the animals’ behavior. Trials were also initiated by a buzz. Monkeys need to fixate on a white spot within a radius of 0.8–1.0° for 100 ms. Then, a red ring (diameter: 3°) appeared for 400 ms as a cue to indicate the location to be attended. The cue appeared at the same location for at least 20 trials. After the cue vanished, four sinusoidal gratings with the identical diameters (2.4°–2.6°), temporal frequency (2 cycles/s), spatial frequency (0.5 cycles/degree), eccentricity (>3°), and contrast (90%) were presented at different locations (refer to Figure 1C) for 1,500–2,500 ms. Three stimuli appeared in the contralateral hemifield (*stimulus locations* 1 and 2: attend-toward; *stimulus location* 3: attend-away near), and the fourth stimulus was placed in the ipsilateral hemifield (*stimulus location* 4: attend-away far). The directions of gratings depended on the preferred directions of the recording neurons. If the preferred directions of the recorded neuronal pair were matched, the other two directions of gratings would be the same. Otherwise, they would be randomly selected and different from each other. To maintain a stable performance (accuracy: >70%), we adjusted the red value of the target stimuli (ranging from 8 to 100) according to the performance curves of the two monkeys (shown in Figure 1B). Animals should make a saccade to this target and fixate it within a radius of 1.5° for 300 ms. Successful identification of the color change within 500 ms was rewarded with a small drop of juice. More rewards were delivered with a shorter reaction time. To assure that the cue directed the monkeys’ focus of attention onto the target spatial location, the color change did not occur at the cued location in 10% of trials in pre-experiment sessions (monkey S, 3 sessions).

### Electrophysiological Recordings

We implemented daily electrophysiological recordings in V1 from a microdrive system with 32 electrodes (Gray Matter Research; travel length: 1.6 cm; interchannel spacing: 1.5 mm; impedances: 0.3–1.5 MΩ at 1 kHz). The recording system was tightly fixed within the chamber. During the insertion procedure, each electrode was controlled by a lead screw independently (125 μm/turn). Based on that, we obtained well-isolated neuronal activities by adjusting the depth of several electrodes.

Neural signals were filtered through a 300 to 4 kHz bandpass, and waveform segments over the threshold were digitized at 40 kHz. We used spike-sorting software (Offline Sorter, Plexon Inc., Dallas, TX, United States) to perform spike sorting offline. Neurons were analyzed further only when their recorded signals were up to our criteria, that is, the proportion of short interspike interval (ISI < 1 ms) was less than 0.2%, waveform shape signal-to-noise ratios (SNRs) were larger than 2.4, and amplitude signal-to-noise ratios (SNRa) were larger than 1.2.

Our dataset included 77 sessions (monkey P, *n* = 40; monkey S, *n* = 37). In a total of 168 well-isolated neurons that met the above criteria, 3 neurons were discarded because they were found to be suppressed by flanking orthogonal stimuli. We selected 83 neuronal pairs (monkey P, *n* = 38; monkey S, *n* = 45) from 165 single units (monkey P, *n* = 80; monkey S, *n* = 85). The horizontal distances between two electrodes recording neuronal pairs simultaneously were 6.22 mm on average (range: 3.35–7.50 mm). Neurons in both monkeys were pooled together to form a single dataset since no differences in tuning properties or attentional modulation of firing rate were observed across neurons recorded from them (all *p* > 0.5, Wilcoxon test).

### Data Analysis

We classified the behavioral results as “correct responses,” “no fixation,” “fixation break,” “early response” (making a saccade before color change), “incorrect responses” (responding to the wrong target), “no response” (failing to respond to target within 500 ms), and “target break” (identifying the correct target but fail to maintain fixation for 300 ms). Our analyses were performed on responses during correct trials only. For each session, we calculated the mean reaction time of correct responses and the detection accuracy (correct responses / [correct responses + early response + incorrect response + no response + target break]). We included recording sessions with at least 20 correct-response trials in each attention condition.

We quantitatively mapped RFs by recording the neuron responses to the Hartley stimuli and using reverse subspace correlation analysis to calculate the spike-triggered averages (STAs) (Ringach and Shapley, 2004). The RF centers of recorded neurons were stable across sessions with eccentricities of 4–6° (shown in Figure 1D, monkey P: left, square; monkey S: right, triangle). The average diameter of RFs across experimental sessions was 1.11° ± 0.44° (range: 0.25°–2.04°), which was much smaller than the distance between the RF centers (2.41° ± 0.52°).

In a 500-ms time window before the color change, we calculated the spike counts in 10 ms bins and fitted them with the Gaussian function. Based on that, we determined the time interval at the half-maximum amplitude of the Gaussian curves (Hu et al., 2021). After that, we calculated the recording neurons’ response linearity (F_1_/F_0_) and defined simple neurons (F_1_/F_0_ > 1) and complex neurons (F_1_/F_0_ < 1) (Skottun et al., 1991).

Spike count correlation (*r*_*sc*_) was calculated as the Pearson correlation coefficient of a neuronal pair’s spike counts evoked by the same stimuli. We counted the spikes for 1 s before the color change in each trial and normalized it into *z*-scores ($z=\frac{X-\mu }{\sigma }$). To avoid the artificial influence of outliers, we subtracted the data points with *z*-scores larger than 3 (Bair et al., 2001; Kohn and Smith, 2005).

Since a spike train consists of sequences of the point process, rather than discretely sampled values of the continuous process, we measured the potential functional connections between recording neurons by Granger causality analysis with a point process framework based on the likelihood approach (Kim et al., 2011). First, we used the conditional intensity function (CIF) to get the likelihood function of the spike train of neuron *i*, including *L_i_* (containing all of the available covariates) and $L_{i}^{j}$ (excluding the spiking history of the neuron *j*). After that, we calculated the reduction in $L_{i}^{j}$ compared to *L_i_* as follows:

$$\Gamma_{i\,j}=\text{l}\,o\,g\,\frac{{\text{L}}_{i}^{j}}{{\text{L}}_{i}}$$

Then, we divided the time interval into *M*_*i*_ non-overlapping rectangular windows and analyzed the Granger causality from neuron *j* to neuron *i*, and the indicator is proposed as follows:

$$\Phi_{i\,j}=-\text{s}\,i\,g\,n\,\left(\sum_{m=1}^{M_{i}}\gamma_{i,j,m}\right)\,\Gamma_{i\,j}$$

where γ_*i*,*j*,*m*_ represents the influence of neuron *j* upon neuron *i* at the mth time window. The sign of $\sum_{m=1}^{M_{i}}\gamma_{i,j,m}$ indicates that it is either an excitatory or inhibitory effect.

### Statistical Analysis

Considering that there are at least 60 trials in each block based on pre-experiment data, we treated the block averages as independent samples (*n* = 29). The paired *t*-tests were applied to test differences in detection accuracy between valid and invalid cues.

In consideration of session-to-session variability, we treated the session averages as independent samples and performed all statistical tests across sessions on formal experiment data. For behavioral data, repeated-measures ANOVAs (rmANOVAs) were used when statistical analysis involved attention conditions as the within-subject factor. For electrophysiological data, χ^2^-test was used to compare the ratio of simple and complex neurons. Two-factor, repeated-measures ANOVA was used to test the changes in *r*_*sc*_ and Granger causality, with attention condition as the within-subject factor and neuronal type as the between-subject factor. The Bonferroni-corrected *post hoc* analysis was applied to evaluate the contrast between different conditions. Statistical comparisons were considered significant at *p* < 0.05. Data were presented as the mean ± SEM.

## Results

We simultaneously recorded pairs of well-isolated neurons from the primary visual cortex of two monkeys while they were performing an attentional detection task. Single-unit activities were recorded by using an implanted microdrive system with 32 electrodes. We carefully chose the optimal stimulus to evoke strong responses of recorded neurons, respectively, according to the neurons’ RF structure and the neurons’ directional selectivity to drifting gratings. The interactions between paired neurons were analyzed by spike count correlations and Granger causality based on the recorded spike trains.

### Behavioral Task and Performance

We used the four alternative forced choices to train monkeys and applied the cued block design to manipulate the focus of the monkeys’ attention. In the attend-toward (AT) condition, the spatial location (*location 1* or *2*) that overlapped one of the recorded neurons’ RFs was cued. In the attend-away (AA) condition, either *location 3* (attend-away near; AAN) or *location 4* (attend-away far; AAF) was cued (Figure 1B). To determine whether the cue effectively guided the animals’ attentional focus, we compared the detection accuracy between trials with valid and invalid cues. We found that the accuracy was significantly higher when target stimuli appeared at the cued location (*t* = 5.083, *p* = 3e-3, paired *t*-test). Since four stimuli had identical size, temporal frequency, spatial frequency, eccentricity, and contrast, we found that there was no significant effect of attention condition on both reaction time (AT = 259.28 ± 2.35, AAN = 254.72 ± 2.49, AAF = 259.92 ± 1.25; *F* = 2.42, *p* = 0.092, rmANOVA) and detection accuracy (AT = 86.01 ± 0.58%, AAN = 86.90 ± 0.68%, AAF = 85.86 ± 0.68%; *F* = 1.14, *p* = 0.321, rmANOVA). Therefore, we concluded that the change of attended locations did not affect the task difficulty and is unlikely to account for any of our physiological results.

### Electrophysiological Recordings and Classification of Neurons

We obtained stable recordings and conveniently isolated neuronal activity from noise due to the design of the recording system. Since the invalid cue might bring confusion to monkeys and prevent them from maintaining attentional focus on the target stimuli, we excluded the data of invalid cue conditions in the neurophysiological analysis. The recording quality was evaluated by the waveform shape signal-to-noise ratio (SNRs) (Kelly et al., 2007) and amplitude signal-to-noise ratio (SNRa) (Hu et al., 2021). The mean SNRs of 165 V1 neurons across all sessions was 5.72 and the mean SNRa was 3.64, showing that the recording quality is suitable for isolating spiking responses of neurons.

**FIGURE 2:**
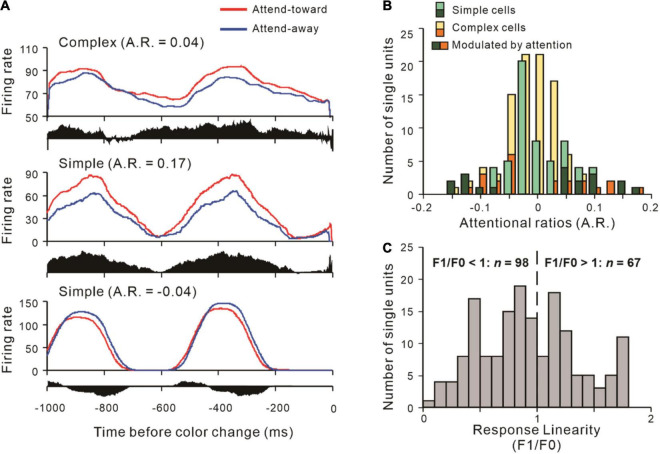
Examples of V1 simple and complex cells and their distributions of attentional ratio. **(A)** Examples of neurons whose firing rates were modulated by spatial attention. The PSTHs show neuronal responses to drifting gratings 1 s before the color change, while the response difference between AT and AA is represented by the black histogram under it. Attentional ratio (A.R.) = (AT – AA) / (AT + AA). A complex (upper) and a simple cell (middle) showed attention-related response enhancement (A.R. > 0), and another simple neuron (down) with spiking response suppression (A.R. < 0). The red solid line indicates the neuronal responses when attention is directed to its RF, while the spiking responses of the attend-away condition are represented by the blue line. **(B)** The number of simple and complex cells with different attentional ratios of firing rate. Several simple (dark green) and complex cells (orange) whose responses were significantly modulated by attention, while others were not (light green: simple cells; yellow: complex cells). **(C)** The distribution of response linearity of 165 V1 neurons across all recording sessions.

To explore how attention modulates the interactions between neural populations, we first calculated the response linearity of recorded neurons, and the distribution of that is shown in Figure 2C. Then, we classified the neurons as simple cells (S, *n* = 67) and complex cells (C, *n* = 98) and found no systematic bias in the distributions (χ^2^ 2.861, *p* = 0.091,χ^2^-test). We examined the modulation of V1 activities by spatial attention at 1 s before the color change. Three representative neurons are shown in Figure 2A, and the distributions of attentional ratios of both simple and complex cells are shown in Figure 2B. After that, we paired the recorded neurons as SS (*n* = 9), SC (*n* = 48), and CC (*n* = 24).

### Effects of Attention on **r**_**s***c*_ Among Neuronal Pairs

**FIGURE 3:**
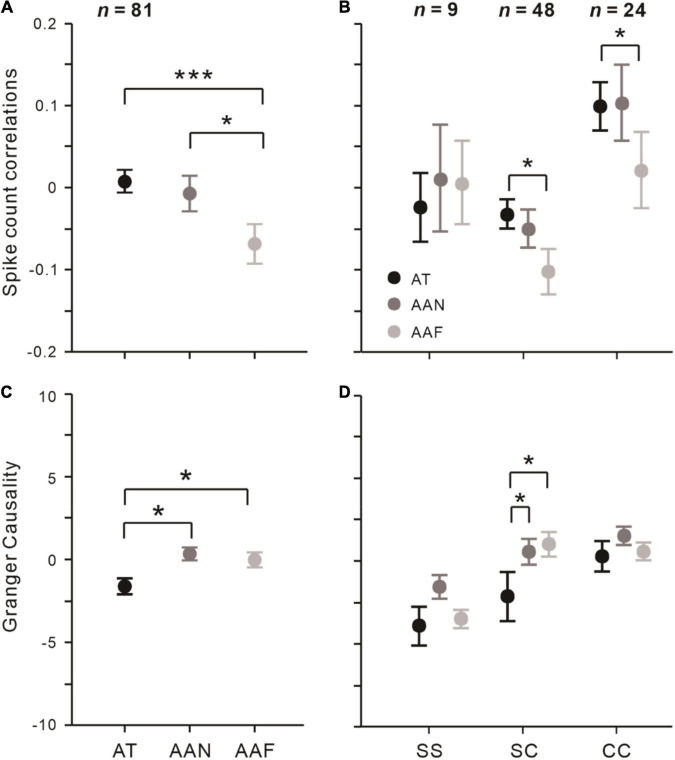
Effects of spatial attention on *r*_*sc*_ and Granger causality among different conditions and groups of neuronal pairs. **(A)** *r*_*sc*_ and **(C)** Granger causality in AT, AAN, and AAF conditions that averaged across all pairs (*n* = 81). **(B)** *r*_*sc*_ and **(D)** Granger causality among SS (*n* = 9), SC (*n* = 48), and CC (*n* = 24) pairs. Error bars represent ± SEM. Repeated-measures ANOVAs and Bonferroni-corrected *post hoc* analysis, *p < 0.05, ***p < 0.001.

We found that attention conditions were significantly modulated *r*_*sc*_ (*F* = 7.470, *p* < 0.001, rmANOVA). As seen in Figure 3A, correlations in the AAF condition (*r*_*sc*_ = –0.066 ± 0.022) was significantly smaller than that in AT (*r*_*sc*_ = 0.011 ± 0.021; *t* = –3.685, *p*_*bonf*_ < 0.001, Bonferroni *post hoc* test) and the AAN (*r*_*sc*_ = –0.006 ± 0.022; *t* = –2.853, *p*_*bonf*_ = 0.015, Bonferroni *post hoc* test), while correlations were not significantly different between the AT and AAN conditions (*t* = 0.832, *p*_*bonf*_ > 0.05, Bonferroni *post hoc* test).

Then, we tested how the type of neuronal pairs modulated the attention effect on *r*_*sc*_. Figure 3B shows that for SS pairs, *r*_*sc*_ did not change significantly under different attention conditions (all *p*_*bonf*_ > 0.05, Bonferroni *post hoc* test); for SC pairs, *r*_*sc*_ in the AT and AAN conditions was significantly higher relative to the AAF condition (AT vs. AAF: *p*_*bonf*_ = 0.011; AAN vs. AAF: *p*_*bonf*_ = 0.013; Bonferroni *post hoc* test) but not different from each other (*p*_*bonf*_ = 1.000, Bonferroni *post hoc* test); and for CC pairs, *r*_*sc*_ was significantly higher in the AT condition than the AAF condition (*p*_*bonf*_ = 0.028, Bonferroni *post hoc* test) and not different among the other conditions (AT vs. AAN: *p*_*bonf*_ = 0.293; AAN vs. AAF: *p*_*bonf*_ = 0.901; Bonferroni *post hoc* test).

### Attentional Modulation of Granger Causality Among Neuronal Pairs

First, we attempted to determine whether the information would flow in a specific direction within the same brain area. We calculated the Granger causality from neuron *j* to neuron *i* across attention conditions (**Φ_i_***_j_*; neuron *i*: RF covered *location 1*, neuron *j*: RF covered *location 2*) and vice versa (**Φ_j_***_i_*). Since there was no significant difference between **Φ_i_***_j_* and **Φ_j_***_i_*in all three attention conditions (all *p* > 0.05, paired *t*-test), we pooled the **Φ_i_***_j_* and **Φ_j_***_i_* together to test the attentional influence.

We also found that Granger causality was significantly influenced by attention (*F* = 3.355, *p* = 0.037, rmANOVA; Figure 3C). Specifically, the AT condition was significantly smaller than the AAN (AT: –1.626 ± 1.290, AAN: 0.586 ± 0.691, *t* = −2.360, *p*_*bonf*_ = 0.020; Bonferroni *post hoc* test) and AAF conditions (0.348 ± 0.689, *t* = –2.106, *p*_*bonf*_ = 0.037; Bonferroni *post hoc* test), while it did not differ between the AAN and AAF conditions (*t* = 0.254, *p*_*bonf*_ = 1.000; Bonferroni *post hoc* test).

Similar with *r*_*sc*_, different types of neuronal pairs varied in attention effects on Granger causality. We found that Granger causality in the AT condition was significantly decreased relative to AAN (*p*_*bonf*_ = 0.031; Bonferroni *post hoc* test) and AAF conditions (*p*_*bonf*_ = 0.012; Bonferroni *post hoc* test) among the SC pairs but not for the other neuronal pairs (Figure 3D).

### Effects of Preferred Directions Differences

**FIGURE 4:**
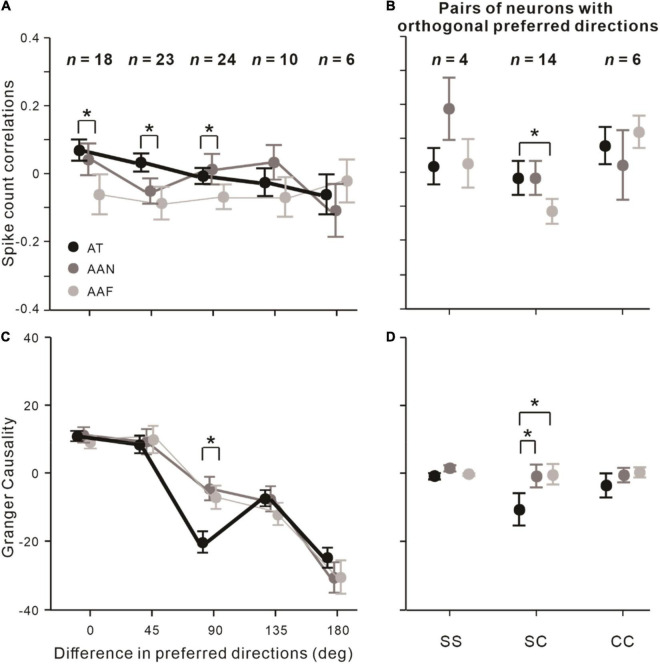
Effects of preferred directions differences on attentional modulations of *r*_*sc*_ and Granger causality between neuronal pairs. **(A)** Effects of difference in preferred directions on *r*_*sc*_. **(B)** *r*_*sc*_ among SS, SC, and CC pairs with orthogonal preferred directions (*n* = 24). **(C,D)** Same as **(A,B)** but analyzed on Granger causality. Error bars represent ± SEM. Repeated-measures ANOVAs and Bonferroni-corrected *post hoc* analysis, *p < 0.05.

According to the procedures to measure the neurons’ preferred directions (see the “Materials and Methods” section), the differences in preferred directions were divided into 0° (same), 45°, 90° (orthogonal), 135°, and 180° (reverse). Then, we examined the influence of the difference in preferred directions on *r*_*sc*_ and on Granger causality across three observed conditions. For *r*_*sc*_ (Figure 4A), we found attention conditions still significantly influenced *r*_*sc*_ when the preferred directions differences were 0° (*F* = 4.109, *p* = 0.025, rmANOVA), 45° (*F* = 4.045, *p* = 0.024, rmANOVA), and 90° (*F* = 3.383, *p* = 0.043, rmANOVA). For Granger causality (Figure 4C), we found that attention conditions affected it significantly, only when the directions preferred by the neurons in the pair were orthogonal to each other (*F* = 3.323, *p* = 0.045, rmANOVA).

Since attention influenced both *r*_*sc*_ and Granger causality in the condition of orthogonal preferred directions, we analyzed whether attention affected *r*_*sc*_ and Granger causality depending on the type of neuronal pairs in that condition. The results showed that *r*_*sc*_ in the AT condition was significantly higher than the AAF condition among SC pairs (*p*_*bonf*_ = 0.021; Bonferroni *post hoc* test; Figure 4B), and Granger causality in the AT condition was significantly decreased relative to AAN (*p*_*bonf*_ = 0.021, Bonferroni *post hoc* test) and AAF conditions (*p*_*bonf*_ = 0.020, Bonferroni *post hoc* test) among SC pairs (Figure 4D).

### Attentional Modulation of Neuronal Interactions Cannot Be Explained by Electrode Distance

Since correlation on a slow time scale (∼1 s) could occur even up to 10 mm (Smith and Kohn, 2008), we explored whether the changes over the distance between electrodes would still exist. The electrode distances of recorded neuronal pairs can be divided into groups of pairs that are less than 5 mm (*n* = 12), between 5 and 6.5 mm (*n* = 15), and more than 6.5 mm (*n* = 54).

**FIGURE 5:**
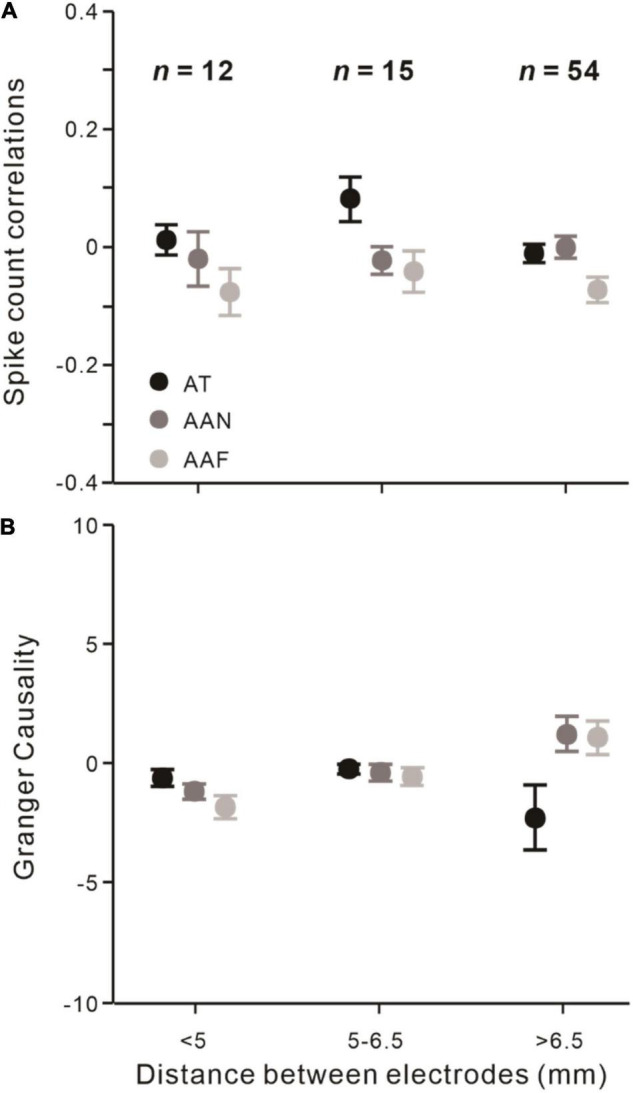
Effects of the distance between recording electrodes among different attentional conditions. **(A)** Effects of different electrodes distances on *r*_*sc*_. **(B)** Same as **(A)** but analyzed on Granger causality. Error bars represent ± SEM. Repeated-measures ANOVAs.

First, we found that*r*_*sc*_ did not significantly change with different distances between electrodes (*F* = 0.389, *p* = 0.855, rmANOVA). The distances of electrodes also did not modulate the attention effect on *r*_*sc*_ (*F* = 1.684, *p* = 0.089, rmANOVA; Figure 5A). Then, we found that Granger causality also did not significantly vary at different electrode distances (*F* = 1.146, *p* = 0.344, rmANOVA). The influence of attention on Granger causality did not significantly change (*F* = 0.722, *p* = 0.703, rmANOVA; Figure 5B).

## Discussion

We aimed to understand how spatial attention influences the interactions between distant neurons in V1 from the perspective of *r*_*sc*_ and Granger causality. We utilized an independent driving system with 32 channels to simultaneously record V1 neuronal pairs in monkeys who performed the attention-demanding color-change detection task. Furthermore, we divided the neuronal pairs into simple and complex cells by response linearity and observe the attentional effects on neural interactions between simple and complex cells. To further understand the effects of attention on neural activity, we analyze the effects of preferred direction differences between pairs on attentional modulation and find spatial attention influence *r*_*sc*_ and Granger causality when pairs have orthogonal preferred directions. It would be worth designing a task that involves more simultaneously recorded V1 neurons with non-overlapping RFs and neurons from higher cortical hierarchies with RFs that cover the former, which is challenging but may provide more information about the mechanism of neural circuits.

### Potential Confounds That Cannot Account for Our Results

First, it has been reported that neuronal interactions decrease with the distance between neurons (Smith and Sommer, 2013; Froudarakis et al., 2014). However, the dependence of *r*_*sc*_ and Granger causality on electrode distances was not observed in our results. One factor that may contribute to our results is that the most simultaneously recording electrodes are 6.71 mm apart (*n* = 48). Also, the distances between electrodes range from 3.35 to 7.5 mm and do not consist of short distances (<1 mm), which is inconsistent with the studies that reported distance effects.

Second, previous researchers reported that the micro-saccades could modulate neuronal activities and enhance neuronal response variability (Gur et al., 1997; Martinez-Conde et al., 2013; Chen et al., 2015; McFarland et al., 2016). To assure that micro-saccades cannot account for our results, we analyzed the eye position across attention conditions during the fixating period before the color change (a 1,000-ms window). We found that the average deviation of eye positions away from the fixation point was about 0.1° in all sessions (mean ± SD = 0.11° ± 0.01°, *n* = 77). Moreover, the direction and frequency of micro-saccades also showed no systematic variation across attention conditions (all *p* > 0.05, paired *t*-test). This shows that fixation eye movements cannot be responsible for our results.

Third, trail-to-trial fluctuations of attentional state contribute to correlated variability (Gu et al., 2011; Ecker et al., 2016; Denfield et al., 2018). First, there are no invalid cues across attention conditions that monkeys need to be ignored, so that attentional fluctuations are minimized in our task. Second, although task difficulty could influence attentional fluctuations (Chen et al., 2017), the difficulty did not change in different attention conditions (see the “Behavioral Task and Performance” section for details). Thus, our results cannot be explained by attentional fluctuations.

Fourth, changes in firing rates across attention conditions also cannot function as an explanation for our results. For spike count correlations, previous studies prove that attention-related changes in spike count correlation do not necessarily result in the same change in firing rates (Zénon and Krauzlis, 2012; Ruff and Cohen, 2014b; Denfield et al., 2018). To further eliminate the influence of firing rates, we also calculated *r*_*sc*_ using the *z*-scores. For Granger causality, it is defined as the natural logarithm of a ratio of residual variances, obtained from two different autoregressive models, which is also not necessarily relevant to the attentional change in the firing rate (Kim et al., 2011).

Furthermore, the previous study found that surrounding stimuli that do not cover the RFs of recorded neurons would affect neurons’ activities (Snyder et al., 2014). Although we cannot eliminate the surrounding suppression in the AT condition, we found that there was no significant difference when the attentional focus was on *locations 1* and *2* in both *r*_*sc*_ (*p* = 0.198, paired *t*-test) and Granger causality (*p* = 0.606, paired *t*-test). It shows that the surrounding suppression makes no difference between the two locations in the AT conditions.

Additionally, we fixed the parameters of gratings in our experiment, which have been reported to affect the responses of neurons in V1. Foster et al. (1985) found that for macaque monkeys’ neurons from V1, the temporal frequency tuning curves with low-pass characteristics peaked even up to 8 Hz and the neural spatial frequency preferences ranged from 0.5 to 8.0 cycles/degree. Low-contrast stimulation would elicit very low firing rates (Ruff and Cohen, 2016; Hembrook-Short et al., 2017). By fitting the size tuning curves, researchers found that gratings with a diameter of 2–3° elicit stronger neuronal responses (Hembrook-Short et al., 2017). Additionally, Denfield et al. (2018) reported that when the eccentricity was smaller than 3°, monkeys would attend to all stimuli simultaneously. Cohen and Kohn (2011) reported that neuronal pairs with very low firing rates would negatively affect the estimate of *r*_*sc*_. Thus, the grating parameters we set up were based on the typical preference of V1 neurons, which would elicit most neurons to respond strongly.

### Comparison With Previous Studies on **r**_**s***c*_ and Granger Causality

The *r*_*sc*_ from our results were negative on average, and its distribution was comprised of both negative and positive ones, which is consistent with studies that explored the neuronal pairs with non-overlapping RFs in the FEF (Cohen et al., 2010) and dlPFC (Leavitt et al., 2013). Although recently reported *r*_*sc*_ are typically small and positive and range from 0.01 to 0.4 (Smith and Kohn, 2008; Gu et al., 2011; Smith and Sommer, 2013; Ruff and Cohen, 2014a; Denfield et al., 2018), the pairs in these studies were overlapped with the same stimulus. Rosenbaum et al. (2017) found that heterogeneous input could change the distribution of *r*_*sc*_ by combining electrophysiological data and computational models. To be more specific, *r*_*sc*_ of pairs in the same neuronal pool were positive on average, while they changed to be strong negative when pairs from different neuronal pools. In our study, we used two different stimuli to overlap anatomically far apart neuronal pairs. Hence, it is not surprising that most pairs were from opposite populations, and most *r*_*sc*_ were negative in our study.

We found that *r*_*sc*_ were lowest in the condition that the attentional focus was directed to the contralateral hemifield of the neurons’ RFs. However, spatial attention-related *r*_*sc*_ decrease was reported in V1 (Smith and Kohn, 2008; Ecker et al., 2010; Ruff and Cohen, 2016), V4 (Cohen and Maunsell, 2009; Mitchell et al., 2009; Ruff and Cohen, 2014a), and MT (Gu et al., 2011; Ruff and Cohen, 2016). First, neurons recorded in the pairs with non-overlapping RFs and different stimuli preferences are considered from different pools in the visual information processing network. The neuronal pairs in our study competed with each other (Granger causality was negative on average). Second, attention would enhance *r*_*sc*_ when the similarity of neurons’ responses to targets (Ruff and Cohen, 2014a) and distractors (Downer et al., 2017) is small. Previous studies reported that neural responses to stimuli were more similar for nearby pairs than for far pairs in visual areas (Kobatake et al., 1998; Erickson et al., 2000; Wang et al., 2000). Since the neuronal pairs from our study are located much farther, the similarity of their responses is much smaller. Third, Granger causality increases from negative to weakly positive when attending away from RFs, which indicates the competing connections between neuronal pairs are diminished. Thus, *r*_*sc*_ could be enhanced by spatial attention in our study.

For the Granger causality, we first determined that the direction of connections within the V1 area was not influenced by spatial attention. The results further support the hypothesis that neurons within the same area are connected recurrently. After that, we focused only on the strength of information flow between two neurons. We found that the Granger causality from our results decreased to negative when the animal attended one of the recorded neurons’ RFs. The results in Granger causality are consistent with previous cross-brain-area studies (Gregoriou et al., 2009; Bosman et al., 2012), which show the attention-dependency enhancement in directional connectivities. The biological mechanism behind the spatial attention-related Granger causality reduction might be the burstiness. Previous studies reported that when spatial attention is directed into the neurons’ RFs, neuronal burstiness would decrease in the visual area (Anderson et al., 2013; Xue et al., 2017). As burst spikes of one neuron are more efficient at directly driving other neurons than temporally dispersed spikes, other studies have suggested that burst spiking could enhance functional connectivities (Bonifazi et al., 2009; Kwan and Dan, 2012; Womelsdorf et al., 2014). Besides, the synchrony of neuronal responses might be another factor. Previous studies explored the effect of covert spatial attention on neuronal synchrony by calculating phase-amplitude coupling (Esghaei et al., 2015), spike-phase coupling (Esghaei et al., 2018), phase coherence (Zareian et al., 2020), and so on. They found that directing spatial attention to the RFs of recorded neurons decreased neuronal synchrony in both the prestimulus period (Zareian et al., 2020) and stimulus presentation period (Esghaei et al., 2015, 2018). The results of our study suggest that the neurons with non-overlapped RFs are inhibitory connected, and this inhibitory connection is diminished when attentional focus shifts away from the neurons’ RFs. Furthermore, such a Granger causality change within the V1 area possibly means more efficient use of neuronal activities. To be specific, the responses of neurons from the same area would carry redundant information, and spatial attention might reduce this redundancy to improve the efficacy of encoding sensory.

### Attentional Influence on Interactions Between Simple and Complex Cells

We found attentional-related enhancement on *r*_*sc*_ and attention-related reduction on Granger causality only among SC pairs, which implied the information integration between these two types of neurons and supported the traditional theory of the hierarchical architecture of simple and complex cells (Hubel and Wiesel, 1962; Yu and Ferster, 2013). The results also suggested that when neurons have non-overlapped RFs and are far apart, attention would influence the interaction between simple and complex cells rather than the interaction within the same type of neurons. It is consistent with the study of Hembrook-Short et al. (2019), who reported that attentional modulation of neural communication was significantly greater for simple–complex pairs.

For *r*_*sc*_, previous studies found that spatial attention would increase functional communication between two different brain areas by increasing the *r*_*sc*_ between neurons (Ruff and Cohen, 2016; Semedo et al., 2019, 2020). Consistent with these studies, the attention-related *r*_*sc*_ increase may indicate the existence of efficient functional communication between simple and complex neurons. For Granger causality, the results show that the competing connectivities between simple and complex cells were larger when attending to one of the neurons’ RFs, consistent with the biased competition theory (Reynolds et al., 1999).

### The Influence of Preferred Directions Differences

Both *r*_*sc*_ and Granger causality show the close relevance to the direction preferences of neuronal pairs (except the *r*_*sc*_ in AAF condition), that is, highest when the direction preferences are similar and decline gradually when they became different. It is consistent with observations from previous studies that *r*_*sc*_ were strongest between neurons with similar preferences and decreased when the preferences of pairs changed to the opposite (Kohn and Smith, 2005; Cohen and Maunsell, 2009; Smith and Sommer, 2013). In addition to *r*_*sc*_, the results of Granger causality supported that neurons were excitatory connected when the preferred direction differences were within 45°, while the neurons were often inhibitory connected when the differences increased (Hata et al., 1988, 1991; Liang et al., 2017). Furthermore, unlike *r*_*sc*_ (attention-related change happens within 90°), we found that attention influences Granger causality only when the recorded neuronal pairs have orthogonal preferred directions. It indicates that when the neurons have similar preferences, attention influences the neurons’ activities by the shared variability between neurons; and when pairs have orthogonal preferred directions, attention would influence the shared variability and connectivities simultaneously.

Taken together, our results suggest that neurons would hold representations of targets and distractors. The effects of spatial attention change with the relative position between the attentional focus and neurons’ RFs, that is, neurons’ functional communication and competing connections would be inhibited when attentional focus shifts away from neurons’ RFs. What is more, we only found a consistent attention-related change in SC pairs, which enriches the architecture theory of simple and complex cells. That is, attention would modulate the communications and connections between simple and complex cells rather than within them, even if neuronal pairs are far apart. Furthermore, we found the effects of the differences in preferred directions on attentional modulation, which indicates attention would prompt neurons to adopt different strategies for different preferred directions’ similarity. Taken together, the present study enriches the understanding of neural mechanisms of attention.

## Data Availability Statement

The raw data supporting the conclusions of this article will be made available by the authors, without undue reservation.

## Ethics Statement

The animal study was reviewed and approved by the Institutional Animal Care and Use Committee of Shanghai Jiao Tong University.

## Author Contributions

QH designed and performed all animal surgery and experiments, made the data analysis, prepared the figures, and wrote the manuscript. ZZ helped with the experiments conducted, made the data analysis, and wrote the manuscript. XS, LL, and XC advised on experimental designs. YC conceived the research and edited the manuscript. All authors contributed to the article and approved the submitted version.

## Conflict of Interest

The authors declare that the research was conducted in the absence of any commercial or financial relationships that could be construed as a potential conflict of interest.

## Publisher’s Note

All claims expressed in this article are solely those of the authors and do not necessarily represent those of their affiliated organizations, or those of the publisher, the editors and the reviewers. Any product that may be evaluated in this article, or claim that may be made by its manufacturer, is not guaranteed or endorsed by the publisher.
